# Towards an automated virtual slide screening: theoretical considerations and practical experiences of automated tissue-based virtual diagnosis to be implemented in the Internet

**DOI:** 10.1186/1746-1596-1-10

**Published:** 2006-06-10

**Authors:** Klaus Kayser, Dominik Radziszowski, Piotr Bzdyl, Rainer Sommer, Gian Kayser

**Affiliations:** 1 UICC-TPCC, Charite, University of Berlin, Berlin, Germany; 2 AGH-UST Krakow, Krakow, Poland; 3 Cairo Consult, Mannheim, Germany; 4 Institute of Pathology, University Freiburg, Freiburg, Germany

## Abstract

**Aims:**

To develop and implement an automated virtual slide screening system that distinguishes normal histological findings and several tissue – based crude (texture – based) diagnoses.

**Theoretical considerations:**

Virtual slide technology has to handle and transfer images of GB Bytes in size. The performance of tissue based diagnosis can be separated into a) a sampling procedure to allocate the slide area containing the most significant diagnostic information, and b) the evaluation of the diagnosis obtained from the information present in the selected area. Nyquist's theorem that is broadly applied in acoustics, can also serve for quality assurance in image information analysis, especially to preset the accuracy of sampling. Texture – based diagnosis can be performed with recursive formulas that do not require a detailed segmentation procedure. The obtained results will then be transferred into a "self-learning" discrimination system that adjusts itself to changes of image parameters such as brightness, shading, or contrast.

**Methods:**

Non-overlapping compartments of the original virtual slide (image) will be chosen at random and according to Nyquist's theorem (predefined error-rate). The compartments will be standardized by local filter operations, and are subject for texture analysis. The texture analysis is performed on the basis of a recursive formula that computes the median gray value and the local noise distribution. The computations will be performed at different magnifications that are adjusted to the most frequently used objectives (*2, *4.5, *10, *20, *40). The obtained data are statistically analyzed in a hierarchical sequence, and in relation to the clinical significance of the diagnosis.

**Results:**

The system has been tested with a total of 896 lung cancer cases that include the diagnoses groups: cohort (1) normal lung – cancer; cancer subdivided: cohort (2) small cell lung cancer – non small cell lung cancer; non small cell lung cancer subdivided: cohort (3) squamous cell carcinoma – adenocarcinoma – large cell carcinoma. The system can classify all diagnoses of the cohorts (1) and (2) correctly in 100%, those of cohort (3) in more than 95%. The percentage of the selected area can be limited to only 10% of the original image without any increased error rate.

**Conclusion:**

The developed system is a fast and reliable procedure to fulfill all requirements for an automated "pre-screening" of virtual slides in lung pathology.

## Background

Tissue – based diagnosis procedures comprise a broad spectrum of techniques. These include, for example, conventional light microscopy images (vessels, cells, nuclei, membranes, extra-cellular substances, etc), visualization of macromolecules and their functions (antibodies, receptors, glycoproteins, etc.), detection of gene arrangements (in situ hybridization), of cytogenetic parameters (point mutations, amplifications, deletions, etc), or live features (cellular movements, etc.) [1-3]. The diagnosis process itself can be distinguished into two different analysis aims: a) the causal conditions and interactions, b) the most effective and appropriate treatment to help the involved patient.

Modern technology permits the digitalization of complete glass slides by so -called slide scanners in a fast and reproducible manner. The obtained image is called a virtual slide, its viewing and analyzing virtual microscopy.

The causative analysis requires distinct theoretical models, is usually embedded in fixed margin conditions, and will not be discussed here furthermore.

A "correct" diagnosis to be used for patients' care possesses the closest association with the most appropriate (and effective) treatment procedure, which can be measured at different stages (times): Prior to the treatment it is called "classic" diagnosis, during the treatment "response" diagnosis, in relation to the outcome of the patient "prognosis" diagnosis, and prior to the outbreak of a disease "risk" diagnosis. The involved biological structures and functions of tissue differ within this development: a "risk" diagnosis is mainly based upon gene arrangements (cancer risk genes), the classical diagnosis mainly upon tissue textures, "response" and "prognosis" diagnosis upon receptors, macromolecules, and gene abnormalities. In a survey according to [4,5] the different diagnosis types and the corresponding tissue examinations are listed in table 1.

**Table 1:** Contribution of different tissue examinations to establishing certain therapy-associated information (diagnosis)

Diagnosis type	Type of tissue analysis			
	Conventional (HE, tissue textures)	Molecule expression (antibodies)	Receptor – binding	Genes

Classic	+++	++	+	-
Prognosis	++	+++	+++	+
Response	+	++	+++	(*)
Risk	-	+	++	+++

(*) with exception of potential germ cell gene therapy vectors

+++ significant, ++ moderate, + minor, - no contribution.

Within the diagnosis procedures certain "ranks" can be distinguished that are related to performance – associated features such as diagnosis "speed", "costs", or human resources (experiences). With exception of the "risk" diagnosis the "classic" diagnosis is a prerequisite for establishing "prognosis" or "response" diagnoses. Based upon these parameters, "conventional" tissue preparation procedures (images obtained from conventionally (HE, PAS, Giemsa, etc.) stained glass slides) form the "gold standard", and are by far the most applied tissue-based diagnosis procedures.

It is, therefore, of theoretical and practical interest, to furthermore analyze the specific conditions of "classic" diagnosis procedure, and to examine the potential benefits of an automated information recognition system associated with conventionally stained glass slides.

## Theoretical considerations

### Information analysis of histological slides

**Figure 1:**
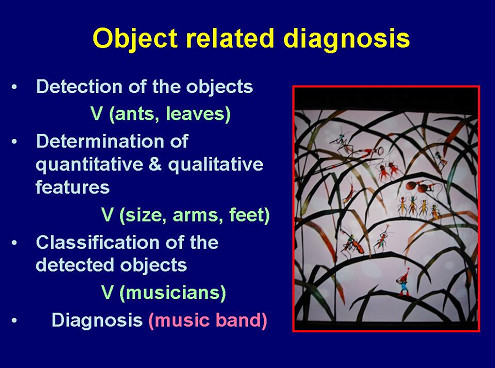
**Scheme of diagnosis algorithm in object – related diagnosis**. Explanation: The original image is divided into a background and an object-related space (right upper and left lower corner). Within the object space object have to be identified by known general object features (ants, leaves). The object features will then be measured and classified according to the feature data set. The complete arrangement will provide the diagnosis.

The information content of light microscopy images obtained from conventionally stained glass slides is composed of two main compartments, namely a) object – associated information, and b) non-object associated information. The detection and classification of object – associated information requires a "division" of the image into an object – related space (compartments), and a non-object – related space (background) [6-10]. The objects searched for are usually "abnormal" events (nuclei, cells, external material, etc.), i.e. objects which display unusual features or which are not present in the analyzed tissue under normal (healthy) circumstances. For example, they comprise cells with alterations in size or internal structures (virus infection), cancer cells, inflammatory cells, or external organisms (bacteria, parasites). The majority of "classic" diagnoses is based upon the detection and correct identification of these "objects": A correct cancer diagnosis requires the correct and error – free proof of cancer cells, that of an active tuberculosis the visualization of tuberculosis bacilli! The basic scheme of object-related diagnosis procedures is given in Figure 1. The first step is to divide the original image into an object and a background image. The second step analyses the objects in relation to their features (cellular and nuclear size, staining intensity, form factor, etc). Of major significance is the procedure of the object – background separation (thresholding), which can be object dependent or not [5,11].

Having identified the objects, their spatial arrangement can possess diagnostic information too, for example in specific growth pattern (granulomas, adenoid growth pattern, epidermoid cellular arrangements, etc.). These features can be analyzed by various techniques, for example by syntactic structure analysis [12-19]. A related graph is constructed which represents the gravity centers of the objects (nodes), a neighborhood relationship (edges), and node/edge related attributes (distances, sizes, integrated optical density, etc.). The procedure allows the definition of new (higher order) objects, if statistical associations (or repeated geometrical figures) can be obtained [4,13,17,18,20].

**Figure 2:**
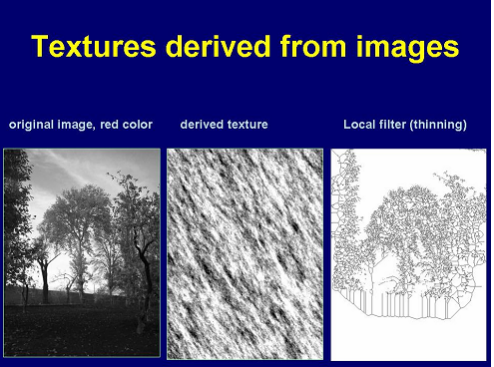
**Original image and derived texture based upon an auto-regressive algorithm**. Explanation: The auto-regression texture analysis yields images of repeated gray value "shadows" that do no longer permit a recognition of the original image in contrast to the application of some local image transformations such as "thinning".

In addition to the described procedures, non-object oriented information can be extracted from a histological image. The underlying representation of image information is usually called texture, and the procedure texture analysis [10,18,21-23]. A texture is a gray value distribution which might possess invariants in image transformation (symmetries). A texture can be analyzed by an autoregressive procedure that computes the gray values of pixels in relation to those of their neighbors. Similar, the same procedure can be applied to create images with artificial textures. A reproducible and invertible texture analysis results in a set of 5 – 6 parameters, and is, therefore, an appropriate tool to compute "similarities" between different images. It can be also used to transform an image into a two dimensional matrix and to compare images with known textures to the diagnostic image [10]. An example of the technique is given in Figure 2.

The application of both object and texture associated diagnosis procedures results in a data set that represents the image as a whole. Thus, these algorithms seem to be useful to analyze virtual slides which represent complete digitized glass slides. However, the digitalization of a complete glass slide creates images measuring several GB in size [1,3,5,24]. Therefore, the question arises whether the diagnosis information content of the complete image can be extracted from included image compartments, and, if yes, what is the obtained accuracy.

### Application of Nyquist's theorem on object oriented information

The image obtained from digitalization of a complete glass slide is called "virtual slide", and commonly measures several GB in size [1,3,5]. It is technically computed by acquisition of several image compartments "patched together" (patch work procedure) [1,5]. The acquisition time measures several minutes or hours, dependent upon the highest wanted resolution. Once such a virtual slide has been acquired, it may be used for numerous purposes including image quantification, storage and retrieval in routine diagnostic work, steering source for automated tissue sampling in tissue micro arrays (TMA), continuous education, etc. The handling of such large data matrices, however, is not easy, and requires fast communicative connections and sophisticated programming. In addition to fast line connections and smart computer solutions, appropriate use of sampling procedures might be useful, might save time and non-necessary efforts. One idea is based upon the principle of tissue – based diagnosis: Once the necessary information needed for diagnosis statement (and confirmation) has been detected, no further efforts are needed, i.e., the diagnostic procedure will be terminated immediately. For example, if tumor cells can be clearly identified in one or several image compartments, there is no need to further analyze the still missing compartments (or the whole image), as this analysis will not affect the diagnosis anymore. Of course, this concept has to be associated with the underlying clinical tasks. For example, the algorithm to clarify a tumor diagnosis can be terminated in a biopsy by identifying the cancer; it has to be continued if resection boundaries have to be investigated too in a surgical specimen.

The decomposition of an image into "diagnosis compartments" and their analysis will, therefore, improve the efficiency of a diagnostic procedure and further allow the calculation of the "risk" of missing an object with diagnostic significance. The risk calculation for object – associated diagnosis depends upon the object number and their size in relation to the sizes of the chosen compartments, as well as upon their sizes and number in respect to the size of the original image. If we consider the probability of an object diagnosis as "original diagnosis frequency" and the compartment division of the original image as "digitalization", we can apply Nyquist's theorem for an optimal adjustment of compartment size to the image. According to Nyquist's theorem the signal to be reconstructed must be sampled with a frequency at least two times greater than that to be reconstructed. In other words, the number of pixels required to classify an object should amount two (in a two dimensional space four) times more than the lower limit of recognition. Similar, the size of the "sampling space", i.e. the diagnosis image compartment must amount four times more than the pixel size of the objects divided by the relative frequency of objects present in the complete image. This assumption is very useful for analysis of histological images, as these images usually contain connected tissue compartments, i.e., numerous cancer cells or bacteria, if correctly taken by the clinician.

Assuming that 10% of the original image (virtual slide) contain diagnostic significant objects, the size of an object measures 100 μm^2^, and an objective of *20 is required to identify the object one would obtain a sample size of 400 μm^2^, which should be repeated N = 1,.2.. 10 times using randomly selected non overlapping samples. If one of the samples contains an object, the procedure can be terminated. An overview of sample size useful for frequently diagnosed histological objects is given in table 2.

**Table 2:** Image volume in relation to objective magnification and optical resolution

**Objective magnification**	4*	10*	20*	40*
**Numerical aperture**	0.2	0.45	0.5	0.75
**Optical resolution (μm)**	1.7	0.75	0.67	0.45
Pixel number*	11765*14704	26667*33333	29851*37313	44444*55556
**Image size**	2.08 GB	11 GB	13 GB	30 GB
**Object size**	1 – 4 MB	1 MB	5 MB	12 MB
Sample size**	8 – 32 MB	8 MB	40 MB	120 MB
**Nuclei**	-	-	*	*
**Cells**	-	(*)	*	*
**Vessels**	*	*	(*)	-
Sample Number***	6 – 25	1375	325	25

* Assuming a slide area (20 * 25 mm); ** Assuming Vv of objects = 0.5; Nyquist's theorem; *** assuming Vv of samples = 0.1

### A survey of sampling procedures

The object – oriented information-extraction requires the identification of objects. The necessary algorithms can be applied to a histological image a) with or b) without additional spatial – associated predefined knowledge. This statement reflects to a random or non-random selection (sampling) of image compartments to be analyzed [5,11,25]. Basically, five different sampling procedures can be distinguished in the analysis of histological slides. They reflect to a) the aim of the image analysis, for example to evaluate the diagnosis information with the highest efficiency, b) to biological features or expected object properties, for example environment independent exhibition of receptors (visualization of macromolecules).

Random sampling does not require predefined information input, and is usually applied for measuring object properties, and the spatial distribution of objects within a tissue. Its counterpart is called stratified sampling, a procedure which either stops when identifying a wanted object (cancer cell), or preferable takes place in certain image areas (for example in close neighborhood to a vessel, at image compartments that display certain specific features, etc.). Both methods, i.e., random sampling or stratified sampling are object – oriented. Thus, they can be performed with a local independent (passive sampling) or local dependent (active sampling) object identification strategy. Active sampling procedures are often necessary in images that visualize macro-molecule expression due to image features that are induced by laboratory conditions.

Finally, quite often "unknown" objects are identified that are difficult to be distinguished from artifacts. They are rare in frequency; might, however, just be common objects which express uncommon features (artifacts). The correct classification of these objects requires an event – and space – related identification of known surrounding objects, and is called functional sampling [5]. The application of stratified either active or passive sampling is most promising for automated extraction of diagnosis – oriented information from a histological image.

### Texture oriented image information

**Figure 3:**
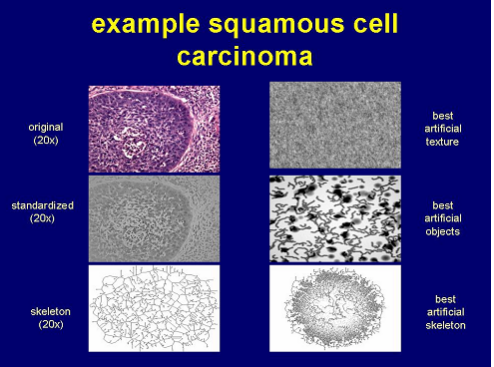
Original histological image, derived standardized and transformed images, as well as best fitting textures and randomly created objects.

In contrast to object – related information, textures can be derived without the division of an image into a foreground (object space) and into a background. Unfortunately, an exact definition of a texture does not exist to our knowledge, neither in general nor in the context of image analysis. Most of the authors use the term "texture" for a general gray value function which can be derived from several repeating and "easy to see" basic image patterns. For example, according to Tamura et al. (1978) a texture can be defined by coarsness, contrast, directionality, line-likeness, regularity, and roughness [23]. Another, more practical and promising approach has been proposed by Voss et al. [10]. The authors use an auto-regression function derived from the analysis of time sequences in order to derive or to create textures. A six dimensional stochastic differential equation describes the correlation of random values (gray values) which are modified by associated coefficients. Figure 3 displays the original image, best fitting randomly computed objects and the calculated texture of a histological image.

The algorithm is basically dependent upon the image size; it becomes, however, quite independent for images of > 2,500 pixels in size (50 * 50 pixels).

The texture synthesis using this auto-regression model and the corresponding derivation of textures from an image to be diagnosed permits a comparison of textures, and the computation of texture similarities. This idea might be appropriate to determine useful diagnostic information based upon image textures.

Naturally, the idea of image analysis by auto-regression algorithms is not limited to the original image, and can be applied to images that have undergone certain transformations of the original image too, such as linear and non-linear local filters (linear shift invariance filtering, Laplace, gradient filtering, etc.).

Reproducible texture analysis does not require an identification of objects, and is, therefore, not associated with object – related information. It is a second, independent approach to extract diagnosis relevant information from a histological image. The approach can be applied to distinguishing between several diagnoses, and is, in addition, able to find new diagnosis items by statistical analysis of the computed features.

### Image trials – methods

**Figure 4:**
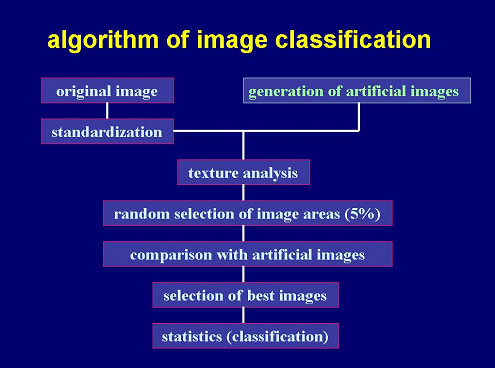
**general scheme of diagnosis algorithm based upon texture analysis only**. Explanation: The algorithm to extract image information starts with a standardization of the image followed by recursive texture analysis and comparison of artificial texture images with those of the training set. The obtained parameters are fed into classification procedures based upon discriminate analysis. This algorithm does not require segmentation procedures.

To prove the discussed theorems, the algorithm for automated extraction of diagnosis – oriented information from conventionally stained histological images was chosen as follows: Still color images were acquired from HE – stained glass slides with a digital camera resulting in an image size 764 * 572 pixels * 8 bits. Non overlapping texture analysis compartments measuring 80 * 50 pixels were randomly defined. Their number was adjusted to the percentage of image space to be analyzed (in this trial 5%). The total image and the image compartments underwent a non-linear filtering (thinning, gradient computation). A linear auto-regression function served for texture analysis of the complete original and transformed image and their randomly selected compartments. For comparison, a corresponding set of artificially created textures of identical image sizes was computed. The total volume fraction Vv of selected compartments was set 5%. The artificially created textures were then compared with the set of textures obtained from images with known diagnoses, and served as classification set. The same procedure was applied to images with unknown diagnoses. The derived textures were compared with those from the classification set, and served for diagnosis classification. The scheme of the applied algorithm is shown in Figure 4.

## Material and results

The trial comprises a total of 996 histological lung images, comprising a training set of 88 cases, and a test set of 808 images. The diagnoses included 349 normal (tumor – free) lung parenchyma, and 647 images showing squamous cell carcinoma, adenocarcinoma, large cell anaplastic carcinoma, and small cell lung cancer. The images were acquired at the microscope objective settings *2.5, *4, *10, *20, *40 which are equivalent to the magnifications (*40, *60, *120, *240, *600). The cases of the learning set were classified using a non-hierarchic discriminate analysis at different classification priorities: The classification priorities reflect to the clinical significance of the diagnosis: in a first step normal lung parenchyma images were separate from tumor images. The second step distinguished between small cell lung cancer and the other three (non-small cell lung carcinoma cell types); the last step in between the three carcinoma cell types (squamous – adeno – large cell).

The texture analysis of a complete image lasted for about 50 ms using a commercially available PC with a tact frequency of 1.2 GB and 512 MB memory size. The self written programs are based upon the visual BASIC – like DIAS language (DIAS, University Jena).

No false positive or negative cases were obtained in differentiating the tumor images from the non-tumor images, the classification accuracy between the different tumor cell types ranged between 96 – 100%. The same result was observed for the other discrimination cohorts. The discrimination accuracy depends upon the chosen magnification: low to moderate magnifications (*60 – *120) displayed the most accurate differentiation between tumor – non- tumor images, in contrast to separate small cell carcinomas from squamous cell carcinomas (*240 – *600). Texture analysis of filtered images was superior to that of the original images.

## Discussion and perspectives

Human performance of tissue – based diagnosis is a quite complex and not really understood procedure. Naturally, image features are recognized, classified, and discriminated in combination with external, non-image data, such as age and sex of the patient. In earlier times, numerous approaches have been undertaken to identify and measure objects and to correlate the obtained features with the tissue-based diagnosis [3,6,12,13,17,25,26]. The automated, feature – related tumor classification was the central aim. The analyzed features included size, shape, chromogen distribution, nucleoli, or more sophisticated second order statistics data [3]. All these trials failed in so far as they did not reach the level of clinical routinely application to our knowledge. More promising was an approach to correlate tissue structures with diagnosis information based upon syntactic structure analysis [15,17,18,21]. This approach revealed some clinical significance, especially in the application of "prognosis" diagnoses [5,21,26].

The development of computer technology offers new perspectives in information extraction of histological images. The prerequisites to develop a successful and accurate system are the analysis of the diagnosis algorithms. The understanding of the "diagnostic procedure" has undergone significant changes too [4,5,27]. Contemporary with the implementation of molecular pathology/genetic methods into routine tissue – based diagnosis our understanding of the diagnostic process itself has altered. Modern pathologists distinguish at least four different types of tissue based diagnoses, which are listed in table 1. As shown in table 1 there is a close association between the technical procedures to be applied and the diagnostic aim. The basic difference between the classical analysis of a histological slide (for example conventional stained slide) and that obtained by application of molecular pathology techniques is based in the visualization of the contained information: The "information extraction" of conventionally stained slides has primarily to recognize "patterns" in contrast to that of molecular pathology data which usually express a "binary information": Antigens, macromolecules, abnormal genes, etc. are either present (expressed) or not.: The visualization of a potential presence of an antigen (antibody) results in a certain color (brown, red) or not, which is correspondent to a binary decision (yes, no).

In addition to the contribution of extra-image features the diagnosis process based upon conventionally stained slides can be separated into two basic procedures, namely a) object dependent, and b) texture dependent.

The object – dependent diagnosis algorithm has to I) divide the image into an object space and a background, II) search for certain objects, III) characterize the objects, and IV) derive the diagnosis – relevant information. Technically speaking, difficulties arise in the definition of the "object space", and the efficient manner to find the objects, i.e., the sampling procedure. As shown in the EAMUS system [8], active stratified sampling is an appropriate method to identify and measure objects present in immunohistochemically stained images. However, the transformation of object related information (object features) into a "conventional diagnosis" cannot be solved in a unique manner according to all the trials that have been undertaken in the past [5].

Texture dependent analysis of histological images has been undertaken by use of graph theory approaches [10,19,24]. In principle, these algorithms define objects as vertices (nodes), use a predefined neighborhood condition (Voronoi, O'Callaghan, or limited distance relationship) to construct the edges, and the features of the vertices (usually nuclei) and their attributes [2,17,18]. These approaches have been reported to be successful for "classic" and "prognosis" diagnosis in lung and breast cancer [2,18]. Obviously, they require similar prerequisites as object dependent diagnosis algorithms, namely the segmentation of the original image into a background and into the object space.

Herein a new approach is presented, a texture based diagnosis algorithm that does not require a segmentation algorithm. The principle idea is the definition and application of a reproducible texture algorithm that is derived from the analysis of time series. This autoregressive model can successfully be applied to create artificial textures, and to reproducible identify image textures [10].

The autoregressive model creates a set of artificial textures and identifies textures of histological images with known diagnosis. Similarly, textures of images with unknown diagnosis are identified too, and compared with the artificial textures, that display the best relation to the textures of images with known diagnosis. The results are promising and convincing: all included cases could be diagnosed without false positive or negative classification. The algorithm requires a minimum image size of 50 × 50 pixels only; i.e., it is useful for image compartments too.

The random selection of non – overlapping image compartments permits an accurate screening of otherwise difficult to handle large sized images (virtual slides). Thus, texture analysis is a promising tool to screen conventionally stained histological slides, and to select those slides for further detailed human analysis that contain diagnosis useful information.
